# Associations Between Antidiabetic Pharmacotherapy and Hearing Thresholds in Adult Hearing‐Impaired Patients With Type 2 Diabetes

**DOI:** 10.1002/prp2.70316

**Published:** 2026-09-10

**Authors:** Francesco Martines, Pietro Salvago, Francesca Vaccaro, Davide Vaccaro, Ginevra Malta, Fabiana Plescia, Stella Cascioferro, Gianluca Lavanco, Fulvio Plescia

**Affiliations:** ^1^ Department of Health Promotion Sciences, Maternal and Child Care, Internal Medicine and Medical Specialties “Giuseppe D'Alessandro” University of Palermo Palermo Italy; ^2^ Section of Audiology, Department of Biomedicine, Neuroscience and Advanced Diagnostics (BiND) University of Palermo Palermo Italy; ^3^ Department of Biological, Chemical and Pharmaceutical Sciences and Technologies (STEBICEF) University of Palermo Palermo Italy

**Keywords:** antidiabetic pharmacotherapy, cochlear pathophysiology, GLP‐1 receptor agonists, hearing loss, hearing thresholds, metformin, pure‐tone audiometry, type 2 diabetes mellitus

## Abstract

Type 2 diabetes mellitus (T2DM) has increasingly been identified as a risk factor for sensorineural hearing loss, whereas the association between different antidiabetic therapies and hearing thresholds remains poorly characterized. This retrospective observational study evaluated 240 patients with T2DM and 105 normoglycemic controls who underwent standardized pure‐tone audiometry at the Audiology Section of the University of Palermo between February 2025 and January 2026. Hearing thresholds were measured using the pure tone average (PTA‐mean) at 0.5, 1, 2, and 4 kHz, corresponding to speech‐relevant frequencies. Multivariable analysis of covariance, adjusted for age, sex, body mass index, and smoking status, showed that subjects with T2DM had significantly higher PTA‐mean values compared to controls (64.4 ± 17.1 dB vs. 56.4 ± 14.8 dB; *p* = 0.007), indicating worse hearing thresholds. Among specific antidiabetic treatments, GLP‐1RAs, metformin, and pioglitazone were associated with lower PTA‐mean values. Furthermore, pharmacological class analysis showed that GLP‐1 receptor agonists and biguanides were associated with more favorable hearing thresholds. These findings suggest that antidiabetic pharmacotherapy may show class‐specific associations with auditory function, supporting the need for prospective studies to evaluate whether hearing outcomes should be considered in the broader management of diabetes‐related comorbidities.

**Abbreviations**
AGEs: advanced glycation end products
AMPK: adenosine monophosphate‐activated protein kinase
ANCOVA: analysis of covariance
Big: Biguanides
BMI: body mass index
CTR: normoglycemic controls
dB HL: decibels hearing level
DPP‐4: dipeptidyl Peptidase‐4 Inhibitors
Gli: glinides
GLP‐1: glucagon‐like peptide‐1
GLP‐1RAs: glucagon‐like peptide‐1 Receptor Agonists
HbA1c: glycated hemoglobin
IQR: interquartile range
PPARγ: peroxisome proliferator‐activated receptor‐γ
PTA: pure tone average
PTA‐mean: pure tone average mean
*R* ^2^
: coefficient of determination
ROS: reactive oxygen species
SD: standard deviation
SGLT2: sodium‐glucose co‐transporter 2
Sul: sulfonylurea
T2DM: type 2 diabetes mellitus
Thi: thiazolidinediones
*β*
: regression coefficients

## 1 Introduction

Diabetes mellitus represents one of the most prevalent chronic metabolic conditions worldwide, affecting an estimated 589 million adults in 2024 and imposing substantial public health burdens [1, 2]. The disease encompasses a heterogeneous group of disorders characterized by hyperglycemia resulting from an absolute or relative defect in insulin secretion and/or reduced insulin sensitivity, with type 2 diabetes mellitus (T2DM) accounting for the vast majority of cases [3]. In Italy, the estimated prevalence of the adult population has grown to approximately 6.6% (approximately 4 million people), with around 87% of patients undergoing pharmacological treatment at specialist centers [4, 5]. The epidemiological landscape has changed notably, with male predominance (6.9%) now exceeding female prevalence (5.7%), revealing broader demographic and lifestyle changes [4, 5]. Annual incidence estimates suggest that 5–7 new cases of T2DM occur every 1000 individuals, with an alarming increasing trend among younger populations [6, 7, 8, 9].

The development of chronic hyperglycemia, typical of T2DM, leads to a cascade of microvascular and macrovascular complications that significantly compromise the quality of life and survival. The well‐known complications related to diabetes include microvascular manifestations such as retinopathy and nephropathy, as well as macrovascular conditions including peripheral vascular and cerebrovascular disease, and myocardial ischemia, all of which represent major contributors to morbidity and mortality [10, 11, 12, 13, 14, 15]. Beyond these traditionally recognized complications, accumulating evidence indicates that a substantial amount of diabetic patients experience progressive sensorineural hearing loss, supporting the concept that the auditory system may represent an additional target of diabetes‐related microvascular and metabolic damage, a complication that has historically received less clinical attention despite its considerable impact on communication, social integration, and psychological well‐being [16, 17, 18, 19].

Diabetes‐related hearing loss was first described by Axelsson and colleagues and is characterized as sensorineural hearing impairment, reflecting primary pathology in inner ear structures rather than conductive mechanisms [20, 21]. The condition typically appears as a progressive, bilateral reduction in hearing perception that preferentially affects speech‐discrimination frequencies. The underlying pathophysiology is multifactorial and consists of several interconnected mechanisms [18, 22]. Cochlear microangiopathy, induced by prolonged hyperglycemia, compromises blood flow to the stria vascularis, a highly metabolically active tissue fundamental for maintaining the ionic gradients necessary for sound transduction [23, 24]. Simultaneously, chronic hyperglycemia generates excessive reactive oxygen species (ROS), promoting oxidative damage predominantly affecting the vulnerable outer hair cells and reducing high‐frequency auditory discrimination [25, 26]. In parallel, chronic low‐grade inflammation and mitochondrial dysfunction further exacerbate cochlear vulnerability by amplifying oxidative stress and impairing cellular energy homeostasis [27, 28]. The accumulation of advanced glycation end products (AGEs) further deteriorates cochlear function through proteolytic and inflammatory pathways [29]. Additionally, diabetic neuropathy and disruption of cochlear ion homeostasis, particularly potassium and calcium dysregulation, contribute to progressive dysfunction of spiral ganglion neurons and hair cells [30]. Emerging evidence also suggests that diabetes‐related neurodegenerative processes may extend beyond the cochlea, affecting auditory nerve fibers and central auditory pathways involved in sound processing [31, 32]. Overall, the interplay between microvascular compromise, oxidative stress, glycation‐related damage, inflammatory processes, mitochondrial dysfunction, and neural dysfunction provides a plausible biological framework linking chronic hyperglycemia to cochlear degeneration and auditory impairment, while also identifying potential mechanistic targets for pharmacological intervention.

Despite the established association between T2DM and sensorineural hearing loss, the precise prevalence is still debated, and the differential effects of specific antidiabetic therapies on auditory preservation have not been systematically characterized in large clinical cohorts [22, 33, 34, 35]. This knowledge gap has important clinical implications, as the selection of antidiabetic agents is typically based on glycemic efficacy, cardiovascular outcomes, and side‐effect profiles, with little consideration given to potential auditory benefits [36].

Recent pharmacological advances have introduced glucagon‐like peptide‐1 receptor agonists (GLP‐1RAs) as effective agents that not only improve glycemic control but also confer cardiovascular and weight‐management benefits through multiple mechanisms, including stimulation of glucose‐dependent insulin secretion, inhibition of glucagon secretion, delayed gastric emptying, and enhanced satiety [37, 38, 39]. Concurrently, metformin, the first‐line agent for T2DM management [40], has emerged as possessing potential neuroprotective properties beyond its metabolic effects [41, 42, 43, 44]. Given the heterogeneous mechanisms of action of antidiabetic drug classes, evaluating their differential impact on hearing function may help disentangle whether auditory preservation is primarily driven by glycemic control or by pleiotropic vascular, anti‐inflammatory, and neuroprotective effects.

This study introduces an integrated evaluation of diabetes status, specific antidiabetic medications, and pharmacological drug classes in relation to objective audiometric thresholds in adults with hearing impairment. Previous research has primarily assessed the association between T2DM and hearing loss, whereas limited clinical evidence has assessed whether distinct antidiabetic therapies are differentially associated with hearing thresholds. By employing standardized pure‐tone audiometry alongside medication‐level and drug‐class analyses, this research establishes a clinically relevant framework for generating hypotheses regarding potential extra‐glycemic auditory effects of antidiabetic pharmacotherapy. Therefore, this investigation aimed to evaluate whether T2DM is independently associated with impaired hearing function and, critically, to assess whether specific antidiabetic pharmacological classes or individual agents are associated with more favorable auditory thresholds in the diabetic population. These objectives are motivated by the clinical imperative to identify therapeutic strategies that simultaneously address multiple dimensions of diabetic comorbidity.

## 2 Materials and Methods

### 2.1 Study Design

This retrospective non‐interventional, observational study analyzed medical records of patients affected by sensorineural hearing loss and visited at the Audiology Section of the University of Palermo between February 2025 and January 2026. The study population comprised two distinct groups: an experimental cohort of 240 individuals with documented T2DM who had undergone comprehensive audiological evaluation and possessed complete documentation of ongoing antidiabetic pharmacotherapy, and a control group of 105 normoglycemic individuals selected from the same clinical archive who had undergone routine pure‐tone audiometry without evidence of fasting glucose abnormalities.

**FIGURE 1:**
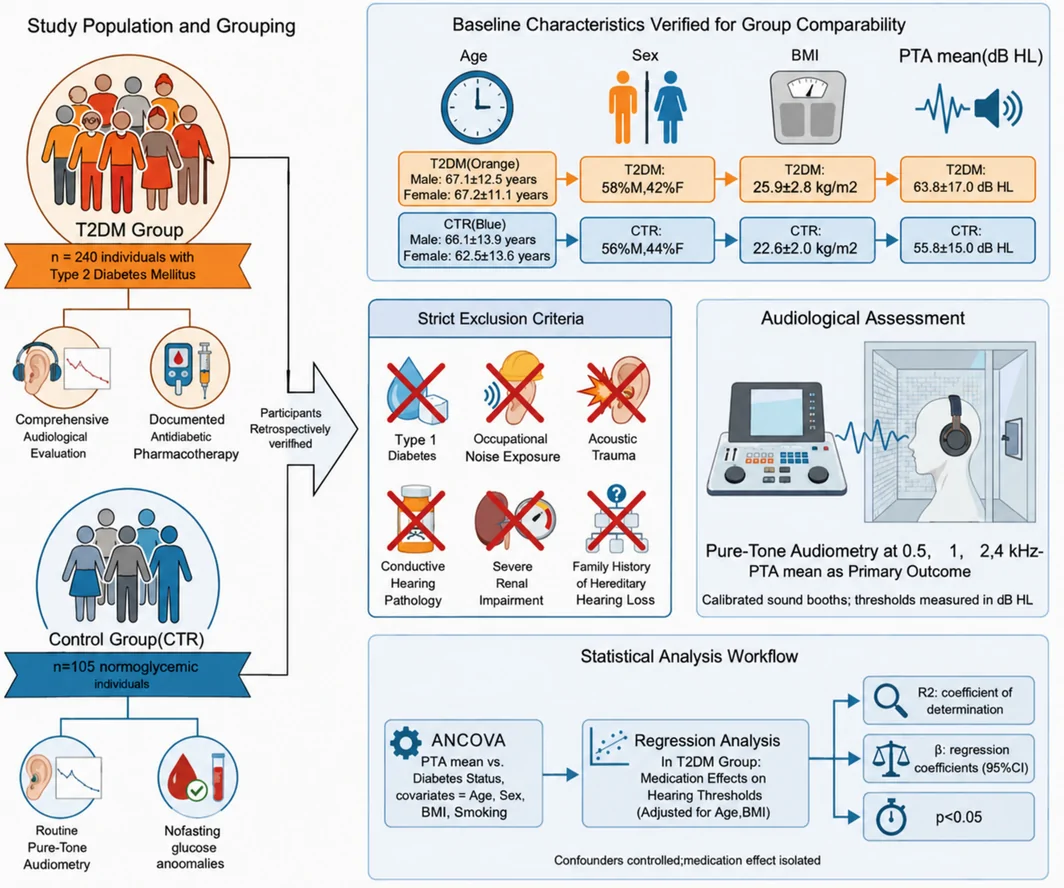
Study design and protocol used to evaluate the impact of T2DM and antidiabetic therapies on hearing function. Image generated using FigureLabs Plus (www.figurelabs.ai).

Strict exclusion criteria were implemented to ensure diagnostic specificity and minimize confounding influences: subjects with type 1 diabetes, occupational noise exposure history, acoustic trauma, conductive hearing pathology (middle ear disease), previous otological procedures, or current ototoxic medication use were systematically excluded. Furthermore, patients with severe renal impairment were excluded to avoid confounding from uremia‐induced auditory dysfunction and electrolyte dysregulation [45]. Subjects with documented family history of hereditary hearing loss or syndromic forms of deafness were also excluded (Figure 1).

Demographic and anthropometric comparability between groups was also verified and is presented in Table 1. All participants provided written informed consent for data processing according to Declaration of Helsinki principles, with institutional protocols ensuring anonymity and data confidentiality.

**TABLE 1:** Demographic and anthropometric characteristics of study participants stratified by diabetes status and sex.

Parameters	T2DM (*n* = 240)	CTR (*n* = 105)
**Male (*n* = 197)**	140	57
Age	67.1 ± 12.5	66.1 ± 13.8
BMI (kg/m^2^)	24.9 ± 2.8	22.6 ± 1.9
PTA medium (dB HL)	64.4 ± 17.1	56.4 ± 14.8
**Female (*n* = 148)**	100	48
Age	67.2 ± 11.1	66.5 ± 13.6
BMI (kg/m^2^)	26.9 ± 2.7	22.6 ± 2.0
PTA medium (dB HL)	63.1 ± 16.8	55.1 ± 15.1

*Note:* This table presents baseline demographic and audiological characteristics for the study population, stratified by diabetes status and biological sex. Data are presented as mean ± standard deviation (SD) for continuous variables. Age is expressed in years. Body mass index (BMI) is calculated as weight in kilograms divided by height in meters squared (kg/m^2^). Pure tone average (PTA‐mean) represents the arithmetic mean of air‐conduction hearing thresholds at speech‐critical frequencies (0.5, 1, 2, and 4 kHz), expressed in decibels hearing level (dB HL).

Abbreviations: CTR, normoglycemic controls; T2DM, type 2 diabetes mellitus.

### 2.2 Audiological Assessment

All subjects included were evaluated through micro‐otoscopy to rule out middle ear pathologies and/or active infections. Pure‐tone audiometry (PTA) was performed by a trained audiologist with a Piano clinical audiometer (Inventis, Padua, Italy) in a soundproof audiometric room. Air conduction was measured without hearing aids using on‐ear TDH‐49 headphones set to 250–8000 Hz; bone conduction was measured using a calibrated bone transducer at 250–4000 Hz. The primary outcome measure was the PTA at speech frequencies (PTA‐mean), calculated as the arithmetic mean of air‐conduction thresholds at 0.5, 1, 2, and 4 kHz frequencies, that are critical for speech intelligibility and widely employed in epidemiological auditory research [46, 47]. In the present study, lower PTA‐mean values were interpreted as indicating relatively better‐preserved hearing thresholds, whereas higher PTA‐mean values indicated greater hearing impairment. For participants with asymmetrical hearing loss, PTA‐mean values were computed separately for each ear and subsequently averaged to provide a globally representative estimate while minimizing ear‐specific variability artifacts [48] (Table 1). This approach provides a sensitive marker of cochlear dysfunction in metabolic and microvascular disorders, making it particularly suitable for detecting diabetes‐related auditory damage.

To ensure the stability of middle ear function, tympanometry was performed using a Clarinet clinical tympanometer (Inventis, Padua, Italy) with a probe frequency of 226 Hz and an air pressure range of −400 to +200 daPa with automatic recording.

## 3 Statistical Analysis

Statistical analyses were performed using Jamovi software (version 2.6.26; Sydney, Australia). To determine whether diabetes mellitus is independently associated with hearing thresholds, univariate analysis of covariance (ANCOVA) was conducted with PTA‐mean as the dependent variable, diabetes status as the categorical independent variable, and age, biological sex, BMI, and smoking status as a priori confounding covariates. This approach isolated the diabetes‐specific effect while accounting for known modulators of age‐related hearing loss.

Afterwards, multivariable linear regression analysis was restricted to the T2DM population to identify associations between individual antidiabetic medications and PTA‐mean values, adjusted for age and BMI.

Diabetes‐specific clinical variables, including diabetes duration, HbA1c, hypertension, cardiovascular comorbidities, smoking intensity, renal function parameters, cumulative drug exposure, treatment duration, and treatment intensity, were not uniformly available in the retrospective records and therefore could not be included in the regression models. Accordingly, the medication‐related analyses were considered exploratory and aimed at identifying associations rather than causal treatment effects.

Individual medications were analyzed first, followed by grouping into pharmacological classes (GLP‐1RAs, biguanides, sulfonylureas, thiazolidinediones, DPP‐4 inhibitors, SGLT2 inhibitors, glinides). Model performance was assessed via *R* ^2^ (coefficient of determination), and multicollinearity was evaluated through variance inflation factor analysis. For all models, non‐standardized regression coefficients (*β*), 95% confidence intervals, and corresponding *p*‐values were computed with *α* = 0.05 as the significance threshold. Residual analysis verified normality of model errors and homogeneity of variance. Effect sizes for significant associations were interpreted clinically with reference to known age‐related hearing loss patterns.

For interpretation of the regression models, negative *β* coefficients were considered to indicate an association with lower PTA‐mean values and therefore relatively better hearing thresholds, whereas positive *β* coefficients indicated an association with higher PTA‐mean values and worse hearing thresholds.

To address whether hearing thresholds differed according to antidiabetic pharmacological class, medications were grouped into GLP‐1 receptor agonists, biguanides, sulfonylureas, thiazolidinediones, DPP‐4 inhibitors, SGLT2 inhibitors, and glinides. Each class was entered into the regression model as an independent categorical predictor, with PTA‐mean as the dependent variable. The analysis was intended to compare drug‐class associations with hearing thresholds rather than to establish causal treatment effects.

The use of ANCOVA allowed us to identify whether diabetes itself is associated with hearing impairment, controlling for major demographic confounders. The subsequent regression analyses restricted to diabetic patients were intended to explore medication‐related associations with PTA‐mean values after adjustment for available covariates. Given the retrospective observational design and the absence of several diabetes‐specific clinical variables, these analyses should be interpreted as exploratory and should not be considered evidence of causal medication effects.

## 4 Results

### 4.1 Type 2 Diabetes and Hearing Function

**FIGURE 2:**
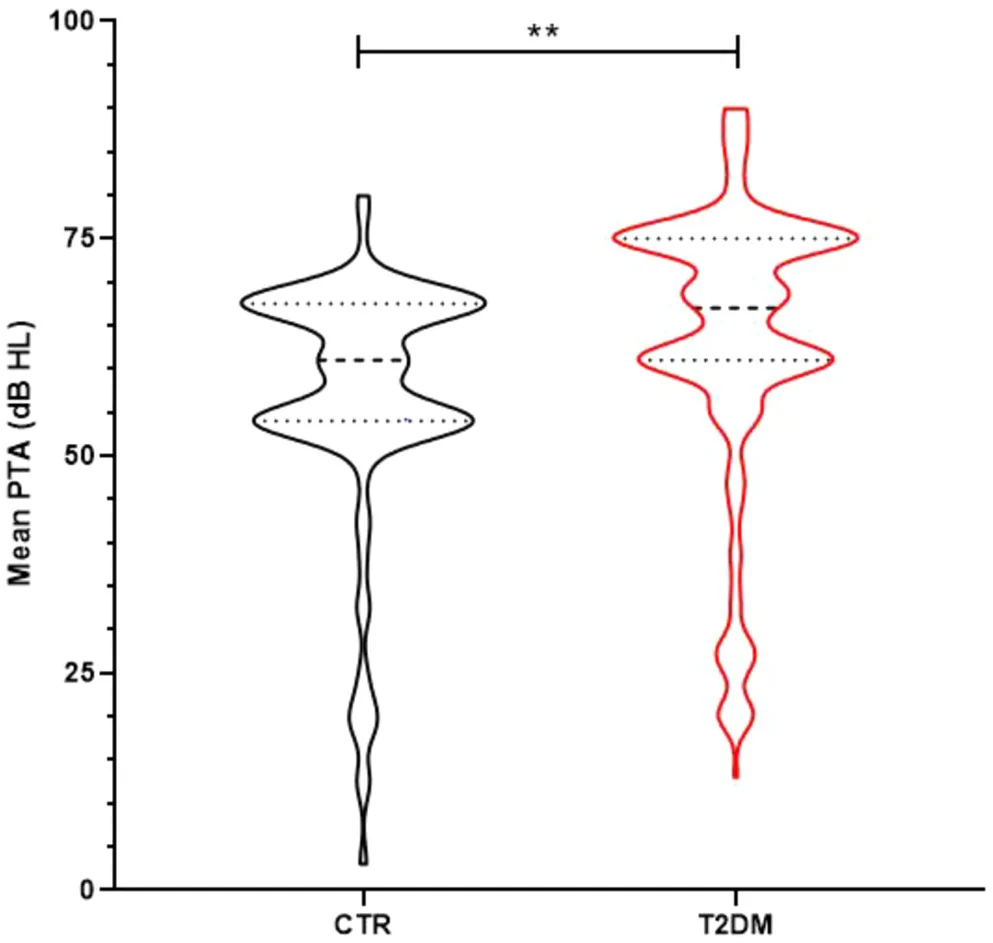
Control group (CTR) is used as the reference category. *p < 0.05; **p < 0.01 vs. CTR.

To evaluate the association between diabetic pathology and hearing function, an ANCOVA was conducted with the mean pure‐tone average (PTA‐mean) as the dependent variable. The model was adjusted for major confounders, including age, biological sex, BMI, and smoking habits. Statistical analysis highlighted a statistically significant effect of the presence of the pathology [*F*_(1,335)_ = 7395; *p* = 0.007], indicating that subjects affected by T2DM have significantly higher hearing thresholds, indicating worse hearing function, compared to the control group, regardless of the other variables considered in the statistical model used (Figure 2). No statistically significant influences emerged for the covariates age (*p* = 0.761), BMI (*p* = 0.854), biological sex (*p* = 0.440) or smoking habit (*p* = 0.582). Furthermore, no statistically significant interactions were highlighted between the variables analyzed within the same group. The nonsignificant association with age (*p* = 0.761) may be explained by the characteristics of the study population, which included only individuals with documented sensorineural hearing loss. Because age‐related auditory impairment was likely already common in both diabetic and control participants, the additional independent contribution of age may have been less apparent in the adjusted model. Therefore, this finding should not be interpreted as evidence that age is unrelated to hearing loss, but rather as a result influenced by the selected hearing‐impaired cohort and the structure of the statistical model.

### 4.2 Association Between Drug Therapies and Hearing Thresholds in Diabetic Patients

Before evaluating the association between antidiabetic therapies and PTA‐mean, the distribution of the primary antidiabetic pharmacological classes in the T2DM cohort was examined. As shown in Table 2, biguanides represented the most frequent pharmacological class, followed by GLP‐1 receptor agonists, sulfonylureas, DPP‐4 inhibitors, glinides, thiazolidinediones, and SGLT2 inhibitors.

**TABLE 2:** Distribution of primary antidiabetic pharmacological classes in the T2DM cohort.

Pharmacological class	*n*	(%)
GLP‐1 receptor agonists	39	16
Biguanides	132	55
Sulfonylureas	21	9
Thiazolidinediones	11	5
DPP‐4 inhibitors	14	6
SGLT2 inhibitors	10	4
Glinides	13	5

*Note:* Data are reported as number and percentage of patients. Each patient was assigned to the primary antidiabetic pharmacological class recorded at the time of audiological assessment. Percentages were calculated using the total T2DM cohort as the denominator (*n* = 240).

Abbreviations: %, percentage of patients; *N*, number of patients.

A multiple linear regression analysis was conducted in the T2DM subpopulation to evaluate the association between different antidiabetic medications and PTA‐mean values. The model was adjusted for the confounding variables, age and BMI. The entered system of variables showed good predictive ability, explaining 29.9% of the total variance in hearing threshold (*R* ^2^ = 0.299).

Among GLP‐1RAs, semaglutide (*n* = 27) and dulaglutide (*n* = 12) were associated with lower PTA‐mean values, indicative of more favorable hearing thresholds (*β* = −27.69; *p* < 0.001; *β* = −28.93; *p* < 0.001). Given the relatively large magnitude of these medication‐level coefficients, these estimates should be interpreted cautiously, as they may be influenced by subgroup size, sparse‐data effects, outliers, or residual unmeasured clinical differences between treatment groups. Lower PTA‐mean values were also observed for pioglitazone (*β* = −33.62; *p* = 0.004) and metformin (*β* = −9.90; *p* = 0.037), suggesting an association between these medications and more favorable hearing thresholds in diabetic patients. No statistically significant differences were highlighted for the other drugs analyzed (*p* > 0.05). Finally, neither age (*p* = 0.439) nor BMI (*p* = 0.415) was a significant factor influencing hearing threshold in this model, suggesting that the observed medication‐related associations persisted after adjustment for these demographic and anthropometric covariates.

### 4.3 Impact of Different Pharmacological Classes on Hearing Function

To directly compare pharmacological classes, drugs were grouped into GLP‐1RAs, biguanides, sulfonylureas, thiazolidinediones, DPP‐4 inhibitors, SGLT2 inhibitors, and glinides. PTA‐mean was then compared across these pharmacological classes using multivariable regression adjusted for age and BMI, with the CTR group used as the reference category. The regression model yielded a multiple correlation coefficient of *R* = 0.227 and a coefficient of determination (*R* ^2^) of 0.0517, indicating that the model explained 5.2% of the total variance in PTA‐mean. This relatively modest explained variance is consistent with the multifactorial nature of hearing function and suggests that pharmacological class represents only one of several factors associated with auditory thresholds.

Despite the limited proportion of explained variance, the analysis showed that GLP‐1RAs and biguanides were significantly associated with lower PTA‐mean values, whereas sulfonylureas, DPP‐4 inhibitors, SGLT2 inhibitors, thiazolidinediones, and glinides did not show statistically significant class‐level associations with PTA‐mean.

In particular, GLP‐1RAs showed the strongest association with lower PTA‐mean values, with a reduction of 9.95 dB compared with the reference category (*β* = −9.95; *p* = 0.003). Biguanides also showed a significant association with lower PTA‐mean values, with a reduction of 4.05 dB (*β* = −4.05; *p* = 0.038). Glinides showed a quantitatively relevant but not fully significant association (*β* = −10.92; *p* = 0.070). Similarly, thiazolidinediones did not show a statistically significant class‐level association (*β* = 5.81; *p* = 0.548), likely due to the limited sample size for this pharmacological class. DPP‐4 inhibitors (*p* = 0.914), SGLT2 inhibitors (*p* = 0.536), and sulfonylureas (*p* = 0.239) were not significantly associated with PTA‐mean values (Figures 3A and 3B).

**FIGURE 3A:**
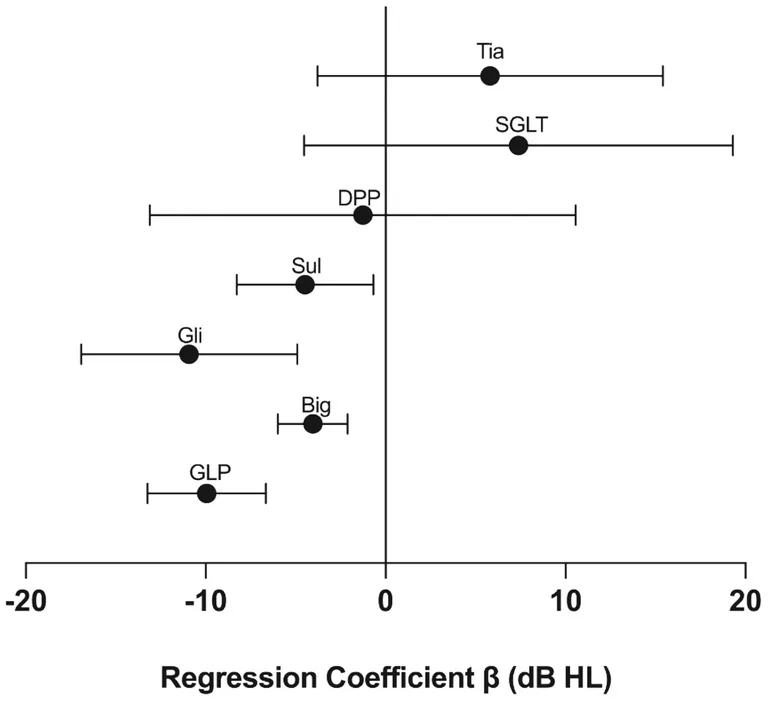
Association between antidiabetic pharmacological classes and hearing threshold. The graph reports the non‐standardized regression coefficients (*β*) and their standard errors (SE) for each class of hypoglycemic drugs, assuming the control group (CTR) as the reference (*β* = 0). Negative *β* values indicate lower PTA‐mean values, corresponding to better hearing thresholds. GLP‐1RAs and Biguanides were significantly associated with lower PTA‐mean values (*p* < 0.05) in the adjusted model. Big, Biguanides; DPP, Dipeptidyl‐peptidase IV inhibitors; Gli, glinides; GLP, glucagon‐like peptide 1 agonist; SGL, sodium‐glucose co‐transporter 2 inhibitors; Sul, sulfonylurea; Thi, thiazolidinediones.

**FIGURE 3B:**
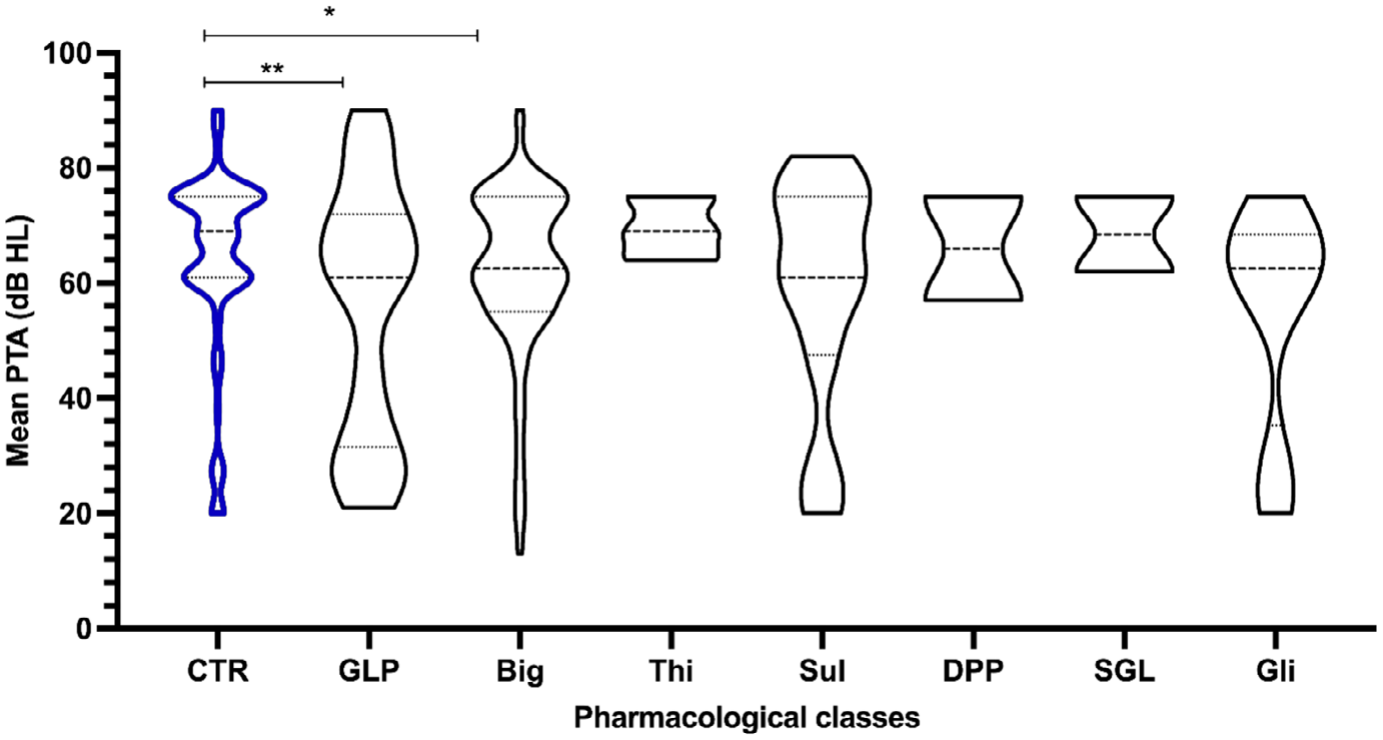
Distribution of hearing thresholds across pharmacological classes compared to health controls. Violin plots illustrate the probability density of the mean pure tone average (PTA) for each group, with the width of the shaded area representing the frequency of data points at that threshold. Inside each violin, the thick horizontal line represents the median, and the dashed lines indicate the interquartile range (IQR). The health control group (CTR) serves as the baseline. *p < 0.05; **p < 0.01 vs. CTR. Big, Biguanides; DPP, Dipeptidyl‐peptidase IV inhibitors; Gli, glinides; GLP, glucagon‐like peptide 1 agonist; SGL, sodium‐glucose co‐transporter 2 inhibitors; Sul, sulfonylurea; Thi, Thiazolidinediones.

Consistent with preliminary analyses, once the drug regimen was included in the multivariate model, neither age (*p* = 0.889) nor BMI (*p* = 0.148) remained statistically significant predictors of PTA‐mean. This suggests that, within the analyzed cohort, antidiabetic pharmacological class was associated with variation in hearing threshold independently of age and BMI. However, given the retrospective observational design and the modest *R* ^2^ value, these results should be interpreted as associations and not as evidence of causal drug effects on hearing function.

## 5 Discussion

The results of the present study provide further insight into the association between T2DM and sensorineural hearing impairment, a relationship that persists after adjustment for established demographic and anthropometric confounders [49, 50]. Moreover, these results highlight the importance of medication‐specific associations with auditory thresholds, suggesting that specific antidiabetic agents, particularly GLP‐1RA and metformin, were associated with lower PTA‐mean values [51]. These observations carry potential important implications for both clinical care and mechanistic understanding of diabetes‐related auditory pathophysiology, suggesting that hearing loss should be recognized as a comorbidity warranting greater consideration for routine audiological assessment [52].

The association between diabetes and hearing impairment observed hereby aligns with studies emphasizing T2DM as an independent risk factor for sensorineural hearing impairment [53, 54]. The magnitude of the observed effect, an 8 dB increase in PTA‐mean comparing diabetics to normoglycemic individuals, is clinically relevant, as a 10 dB increase in auditory thresholds is perceptually equivalent to halving perceived loudness [55]. The persistence of this association across sex and the failure of demographic covariates to explain the effect support the interpretation that the diabetes‐auditory dysfunction link reflects underlying disease‐related mechanism rather than solely residual confounding [56]. This observation should prompt increased clinical awareness of hearing impairment in diabetic populations and suggests that audiological assessment might constitute a useful adjunctive screening component within comprehensive diabetes care [57]. Overall, these findings are consistent with previous epidemiological and clinical studies reporting an association between T2DM and sensorineural hearing impairment. In addition, our results extend the existing literature by suggesting that hearing thresholds may differ according to antidiabetic medication use and pharmacological class. While the association observed for metformin is in line with emerging clinical evidence, the association between GLP‐1RA use and lower PTA‐mean values should be considered novel and hypothesis‐generating, requiring confirmation in prospective studies.

An interesting finding of this research concerns the associations between GLP‐1RA therapy and lower PTA‐mean values. The substantial magnitude of GLP‐1RA‐associated coefficients, corresponding to reductions in PTA‐mean of approximately 28 dB for individual agents (semaglutide and dulaglutide) and 9.95 dB at the class level, is particularly noteworthy, although these estimates should be interpreted cautiously given the observational design of the study. Nonetheless, these associations are biologically plausible considering the multiple intracellular pathways activated by GLP‐1 signaling that are relevant to cochlear homeostasis [58]. GLP‐1RA agents enhance mitochondrial function, suppress inflammasome activation and pro‐inflammatory cytokine production, reduce ROS generation, and improve endothelial function [59, 60]. The anti‐inflammatory and antioxidant properties of GLP‐1RAs are particularly relevant to diabetes‐related hearing loss, which fundamentally involves oxidative stress and microvascular inflammation within the cochlea [61, 62]. Furthermore, GLP‐1RAs promote cardiovascular and metabolic optimization that may secondarily preserve cochlear perfusion and nutrient delivery. Taken together, these complementary mechanisms likely explain the associations with lower PTA‐mean values observed [63, 64, 65].

The association identified for metformin, though more modest than that observed for GLP‐1RAs, nonetheless warrants consideration of the possible mechanisms underlying this effect. Metformin is recognized as a pleiotropic agent whose cellular effects extend substantially beyond insulin secretion enhancement [66]. Emerging evidence indicates that metformin activates adenosine monophosphate‐activated protein kinase (AMPK), a metabolic sensor that regulates mitochondrial biogenesis, oxidative stress homeostasis, and inflammatory responses [67, 68]. Metformin‐induced AMPK activation may thereby ameliorate the oxidative damage and microvascular dysfunction that characterize diabetes‐related cochlear pathology. Additionally, metformin's capacity to improve systemic metabolic flexibility and enhance cardiovascular outcomes may secondarily benefit auditory tissues that are sensitive to metabolic disruption and ischemia [69, 70]. The persistence of metformin's association after statistical adjustment suggests that its potential auditory benefits may not be solely mediated by glycemic control, although this could not be directly assessed in the present analysis [71, 72].

The association identified for pioglitazone at the individual medication level, though not reflected in the pharmacological class analysis, likely due to limited sample size, aligns with emerging literature regarding thiazolidinedione benefits in microvascular disease. Thiazolidinediones enhance insulin sensitivity through peroxisome proliferator‐activated receptor‐γ (PPARγ) signaling, which suppresses macrophage‐driven inflammation and promotes anti‐inflammatory macrophage polarization [73, 74]. Additionally, PPARγ activation improves endothelial function and reduces atherosclerotic burden, potentially enhancing cochlear microvascular perfusion [75]. However, the nonsignificant class‐level effect suggests that thiazolidinedione benefits, if present, may be less consistent or more context‐dependent than those of GLP‐1RAs or metformin, possibly reflecting individual heterogeneity in patient responsiveness or limited representation within the analysis.

Conversely, the absence of significant associations with lower PTA‐mean values for sulfonylureas, DPP‐4 inhibitors, and SGLT2 inhibitors is informative. Sulfonylureas promote insulin secretion through ATP‐sensitive potassium channel closure in pancreatic beta cells, without exerting relevant anti‐inflammatory or antioxidant effects, which may explain the lack of auditory benefit. While DPP‐4 inhibitors and SGLT2 inhibitors possess recognized cardiovascular benefits [76], these agents do not specifically address the oxidative stress and inflammatory mechanisms most centrally implicated in diabetes‐related hearing loss, thereby explaining their null auditory associations [62]. This differential pattern across medication classes supports the hypothesis that lower PTA‐mean values may be related to mechanisms involving oxidative stress suppression and microvascular inflammation reduction rather than to glycemic control per se, since all studied agents effectively reduce blood glucose [77, 78].

These findings should be interpreted considering some limitations. The retrospective observational design does not allow firm causal conclusions regarding the association between antidiabetic pharmacological treatment and hearing thresholds. Residual confounding from unmeasured factors, including diabetes duration, HbA1c, glycemic control, obesity severity, hypertension, cardiovascular comorbidities, smoking intensity, renal function parameters, cumulative drug exposure, timing of treatment initiation, treatment duration, and the presence or severity of microvascular complications cannot be excluded. Therefore, the observed medication‐related associations may partly reflect underlying clinical differences between treatment groups rather than direct pharmacological effects on auditory function. In addition, because longitudinal audiometric follow‐up was not available, this study could not assess true hearing deterioration or preservation over time. Therefore, hearing preservation was defined operationally as lower PTA‐mean values at the time of audiological assessment, and the findings should be interpreted as cross‐sectional associations rather than evidence of slowed auditory decline. Furthermore, hearing assessment relied on pure‐tone audiometry, which, while well established for evaluating cochlear function, does not capture retrocochlear or central auditory processing, nor more complex hearing abilities. Because the study included only hearing‐impaired patients, the range and distribution of hearing function was restricted, potentially explaining the unexpected non‐association of age with hearing thresholds. Medication use was assessed at a single time point, without detailed information on duration, adherence, or dosage, which may have influenced the observed associations. In addition, classification by primary antidiabetic pharmacological class may not fully capture the complexity of combination therapy regimens. Finally, as shown by the subgroup distribution of pharmacological classes, some treatment groups included relatively small numbers of participants. Therefore, estimates for less represented classes and individual medications, particularly those showing large coefficients, should be interpreted cautiously, as they may be more sensitive to sparse‐data effects, outliers, or residual confounding. These findings should therefore be considered exploratory.

Despite these limitations, the present findings may have relevant clinical and research implications. If confirmed in prospective investigations, the associations observed for GLP‐1RAs and metformin may provide a rationale for including audiological outcomes in future studies of diabetes‐related comorbidities [79]. However, the current evidence is insufficient to support changes in antidiabetic prescribing based on hearing outcomes alone. Future studies should therefore evaluate auditory measures alongside traditional metabolic and cardiovascular endpoints to clarify whether antidiabetic treatment patterns are independently associated with hearing‐related outcomes [80]. In addition, the mechanistic pathways proposed herein, particularly oxidative stress, microvascular inflammation, and endothelial dysfunction, should be tested in appropriately designed experimental and translational studies examining cochlear tissue directly, potentially yielding insights relevant to both diabetes‐related and age‐related hearing loss [81].

From a clinical perspective, these findings may increase awareness of hearing impairment as a relevant comorbidity in patients with T2DM, particularly in older adults and in patients with long‐standing disease or additional microvascular risk factors. The results do not currently support changes in antidiabetic prescribing solely on the basis of auditory outcomes. However, they suggest that audiological monitoring could be considered as part of comprehensive diabetes care, especially in patients reporting hearing difficulties. In addition, the observed associations between specific antidiabetic therapies and lower PTA‐mean values provide a rationale for future prospective studies designed to evaluate whether auditory outcomes may represent an additional extra‐glycemic endpoint in diabetes management. A multidisciplinary approach involving diabetologists, audiologists, and primary care physicians may help identify hearing impairment earlier and improve quality‐of‐life outcomes in this population.

## 6 Conclusion

This retrospective observational study found that T2DM was associated with higher PTA‐mean values and worse sensorineural hearing thresholds compared with nondiabetic controls, even after adjustment for age, biological sex, BMI, and smoking status. Notably, this investigation showed that specific antidiabetic pharmacologic agents and classes were associated with lower PTA‐mean values and relatively more favorable auditory thresholds, suggesting that auditory outcomes may represent an additional area of interest in the broader evaluation of diabetes‐related comorbidities. In particular, GLP‐1RAs and metformin were associated with lower PTA‐mean values in the present cohort; however, these findings could be interpreted as exploratory associations rather than evidence of causal pharmacological effects on auditory function. These results support the recognition of diabetes‐related hearing loss as a clinically relevant comorbidity and suggest that the observed associations between antidiabetic medications and auditory thresholds warrant confirmation in prospective studies.

In conclusion, this investigation positions hearing loss within the broader framework of clinically relevant diabetes‐associated complications and highlights those auditory outcomes may deserve greater attention in future studies of comprehensive diabetes care, alongside glycemic and cardiovascular endpoints. This dimension of diabetic care has historically received insufficient clinical and research attention. The pathways identified merit further investigation through appropriately designed prospective and mechanistic studies to clarify whether antidiabetic treatment patterns are independently associated with auditory trajectories and to inform evidence‐based approaches to comprehensive diabetic management that address the full spectrum of patient health and well‐being.

## Author Contributions

**Francesco Martines:** conceptualization, project administration, data curation, investigation, methodology, resources. **Pietro Salvago:** methodology, supervision, validation, writing – review and editing. **Francesca Vaccaro:** data curation, validation. **Davide Vaccaro:** data curation. **Ginevra Malta:** data curation, validation. **Fabiana Plescia:** formal analysis, data curation. **Stella Cascioferro:** data curation. **Gianluca Lavanco:** investigation, writing – review and editing, formal analysis. **Fulvio Plescia:** conceptualization, project administration, supervision, writing – original draft, writing – review and editing, funding acquisition.

## Funding

This research was funded by the University Research Fund—FFR 2025 to Fulvio Plescia.

## Ethics Statement

Given the retrospective and non‐interventional nature of the study, formal approval by an institutional ethics committee was not deemed necessary. The study was conducted using anonymized data, and informed consent was obtained from all participants at the time of hospital access. In accordance with World Health Organization guidance for health research, studies based exclusively on anonymized retrospective data may not require review by a local ethics committee (https://extranet.who.int/kobe_centre/sites/default/files/pdf/WHO%20Guidance_Research%20Methods_Health‐EDRM_6.4.pdf).

## Consent

Informed consent was obtained from all the subjects involved in the study.

## Conflicts of Interest

The authors declare no conflicts of interest.

## Data Availability

The datasets are not publicly available. Anonymized data may be provided upon request by Fulvio Plescia.
